# Effortful control and expressive language in deaf or hard-of-hearing children: The contributions of caregiver language and parenting stress

**DOI:** 10.1017/S0305000925100469

**Published:** 2026-01-12

**Authors:** Kristina Bowdrie, Rachael Frush Holt, William Kronenberger

**Affiliations:** 1 Case Western Reserve University and Department of Speech and Hearing Science, The Ohio State University, Columbus, Ohio, USA; 2 Department of Otolaryngology - Head and Neck Surgery, Indiana University, Indianapolis, Indiana, USA; 3 Department of Psychiatry, Indiana University School of Medicine, Indianapolis, Indiana, USA

**Keywords:** effortful control, parental input, hearing loss, parenting stress, expressive language, temperament

## Abstract

Caregiver–child interactions reflect an important dynamic that supports spoken language development in deaf and hard-of-hearing (DHH) children. This study examined how child effortful control interacts with caregiver language and parenting stress to affect child expressive language. Fifty-nine DHH children (mean age = 5;9) and their primary caregiver participated in a play interaction where expressive language was measured. Caregivers completed questionnaires measuring child effortful control and parenting stress. When caregivers used higher quality language, DHH children demonstrated stronger expressive language regardless of effortful control level compared to when caregivers used lower quality language. Additionally, a trend suggested DHH children with higher effortful control showed stronger expressive language skills when parenting stress was low. However, this trend was not observed when caregivers reported greater levels of parenting stress. These findings support the need to investigate caregiver characteristics that support DHH children in leveraging their inherent regulatory abilities to achieve better language outcomes.

## 1. Introduction

Studies examining spoken language outcomes in children who are deaf or hard-of-hearing (DHH) have identified important and significant contributions from the family environment (Dall et al., 2022; Holt et al., 2020), including influences from caregivers (Yoshinaga-Itano et al., 2020). For example, better spoken language outcomes in DHH children generally are observed when their caregivers provide higher quantity and quality of linguistic input (Ambrose et al., 2015; DesJardin & Eisenberg, 2007; Nittrouer et al., 2020). Additionally, caregiver attributes, such as levels of parenting stress and sensitivity, can influence spoken language development in DHH children (Blank et al., 2020; Quittner et al., 2010; Sarant & Garrard, 2014). While there has rightfully been a heavy emphasis in the literature on the influence of parents on DHH children’s language development, less attention has been given to the dynamic processes that take place between caregivers and DHH children within the microsystem of the family environment. Thus, the purpose of the current study is twofold: (1) to examine the interactive effect of DHH children’s inherent regulatory skills (i.e., effortful control) and caregiver language input on DHH children’s expressive language and (2) to investigate the moderating influence of another caregiver characteristic that influences caregiver–child dynamics (i.e., parenting stress).

### 1.1. Effortful control and language development

Temperament is the product of biological processes that underlie self-regulatory abilities and emotional reactivity to stimuli and remains relatively stable across the lifespan (Rothbart & Sheese, 2007). The constructs used to define these inherent regulatory and reactive abilities vary in the literature (Shiner et al., 2012). However, common and well-accepted temperament constructs used to describe regulatory abilities and positive and negative reactivity include effortful control, surgency-extraversion, and negative affectivity, respectively (Putnam & Rothbart, 2006; Rothbart & Sheese, 2007). Effortful control refers to the ability to leverage one’s inherent regulatory capacities to wilfully adapt attention, behaviour, and emotions (Eisenberg et al., 2004; Rothbart et al., 2003). Surgency-extraversion reflects tendencies to respond to novel stimuli and situations in a positive manner, while negative affectivity reflects negative responses to novel situations (Rothbart & Sheese, 2007). Positive emotional states associated with surgency-extraversion (e.g., elation) are thought to be associated with activation of approach systems while negative emotional states (e.g., fear) are associated with defence systems (Rothbart & Sheese, 2007).

Higher levels of effortful control in children are thought to support language learning through successful regulation of emotional reactivity and behaviours, which conserves cognitive resources that can instead be allotted to attentional and other cognitive processes that allow for language learning (Bloom, 1993; Rothbart et al., 2003). In addition, increased attention in infants and children can prompt more opportunities for language input from caregivers, which also supports language learning (Masek et al., 2021). Positive and negative emotionality have been linked to language development in younger children through eliciting caregiver interactions and responsivity (Spinelli et al., 2018). However, in paediatric populations experiencing typical development, language becomes more resilient to influences of temperament beyond toddlerhood as language development begins to peak during the school-age period (Conture et al., 2013; Spinelli et al., 2018).

***Effortful control and language development in DHH children***. DHH children can experience delays and deficits in many areas of neurocognition, including executive functioning, relative to children without hearing loss or developmental delays (McCreery & Walker, 2022). Additionally, DHH children with better executive functioning skills generally have better spoken language outcomes (Jamsek et al., 2022). Given the associations between executive function and spoken language in DHH children, it can be expected that temperament, or the biological capabilities that precede later neurocognitive processes, may also be associated with spoken language in this clinical population.

The research exploring the relation between effortful control (i.e., a biologically based regulatory capacity) and spoken language is limited in DHH children. However, there is evidence that DHH children may show lower levels of effortful control compared to their peers with typical hearing. For example, DHH toddlers (age 1;6–3;0) with prelingual deafness who used either cochlear implants (CIs) or hearing aids (HAs) showed lower levels of effortful control as rated by caregivers using the Early Childhood Behaviour Questionnaire–Short Form (ECBQ-SF; Putnam et al., 2006) compared to their peers without hearing loss (Castellanos & Houston, 2024). These results also were observed in an older sample of DHH children (age 3;0–7;11) who used CIs and HAs, in which caregivers rated DHH children lower on effortful control via the Child Behaviour Questionnaire–Short Form (CBQ-SF; Putnam & Rothbart, 2006) compared to caregivers of children with typical hearing (Bowdrie et al., 2022). In addition, while the effects of temperament on language in children experiencing typical development begins to taper off by the school-age period (Conture et al., 2013; Spinelli et al., 2018), effortful control appears to remain important specifically for receptive language in school-aged DHH children (Bowdrie et al., 2022, 2023). Previous work has shown that DHH children with greater effortful control showed better receptive language skills compared to DHH children with lower levels of effortful control. There has not been strong evidence that other domains of temperament, including negative affectivity and surgency-extraversion, are significant contributors to language development in school-aged DHH children (Bowdrie et al., 2022).

***The Influence of caregivers on the relation between child effortful control and child spoken language in DHH children***. In many cases, caregivers are the most proximal language model for children. Indeed, for DHH children – who are at risk for inconsistent auditory input even with sensory aids – increased quality (e.g., high-level facilitative language techniques) and quantity of caregiver language is associated with better language skills (Ambrose et al., 2015; Dirks et al., 2020; Moeller & Tomblin, 2015; Nittrouer et al., 2020; Walker et al., 2019). Moreover, as discussed in the previous section, DHH children with greater inherent capacities for self-regulation, such as effortful control, show better language comprehension (Bowdrie et al., 2022, 2023). It is possible that DHH children with greater inherent regulatory abilities have greater cognitive resources available to attend to language provided by caregivers, thereby promoting better language outcomes. Another possibility is that DHH children with greater regulatory capacities appear more attentive to caregivers during social interactions, which can prompt more opportunities for language learning for DHH children. It should also be considered that both explanations contribute to enhanced language outcomes in DHH children who have better self-regulation.

Ultimately, language learning within the family environment reflects a dynamic and transactional exchange between caregivers and children. Work by Bowdrie et al. (2023) was the first to our knowledge to examine interactions between child temperament and caregiver language and their effect on language in school-age (i.e., 3;0–7;11 years) children who are DHH. Specifically, Bowdrie et al. (2023) investigated how effortful control related to language in DHH children depending on the different levels of language input provided by caregivers. Caregivers’ language was quantified using mean length of utterance in morphemes (MLU, i.e., syntactic complexity) and number of different words (NDW, i.e., lexical diversity) used during a semi-structured, dyadic play interaction. They found that when caregivers used low to moderate levels of lexical diversity, only DHH children with better effortful control achieved better language comprehension. However, when caregivers used high levels of lexical diversity, similar language comprehension skills were observed for DHH children with higher and lower levels of effortful control. Similar trends in terms of the interactive effects of child effortful control and caregiver syntactic complexity on receptive language were observed, though these results were only marginally significant. These results suggest that (1) higher effortful control may enable DHH children to achieve better language comprehension skills in environments with limited lexical input and (2) increased lexical input from caregivers can potentially help buffer against poor language comprehension outcomes in DHH children with lower levels of effortful control.

***The influence of parenting stress on the relation between child effortful control and child spoken language in DHH children.*** Given that caregiver language is such a robust predictor of child language outcomes, it is important to also consider caregiver characteristics that can potentially shape the quality and quantity of linguistic input. Parenting stress, for example, is known to affect interactions between children and their caregivers. Parenting stress captures the subjective level of distress related to the challenges of parenting that can affect caregiver–child interactions (e.g., Abidin et al., 2006; Deater-Deckard, 2008). It is thought to be separate from, but can be influenced by, other forms of parental stress that caregivers may experience such as hardships related to employment, finances, and other life events (Deater-Deckard, 2008; Ríos et al., 2022). Increased parenting stress is associated with authoritarian parenting styles, reduced responsiveness, and depression and anxiety in caregivers (Fang et al., 2021; Ward & Lee, 2020), all of which can affect opportunities for language learning in DHH children. Investigating the role of caregiver characteristics such as parenting stress provides the opportunity to reveal underlying mechanisms linking caregiver language with broader parenting demands and their impact on DHH children’s language outcomes.

Emerging research has begun to explore the role of parenting stress on language development in DHH children. For example, despite caregivers of DHH children not showing any differences in overall levels of parenting stress compared to caregivers of children with typical hearing, Blank et al. (2020) found that school-aged DHH children showed suboptimal spoken language comprehension when caregivers reported greater parenting stress on the Parenting Stress Index–Fourth Edition Short Form (PSI-4-SF; Abidin, 2012), a general measure of parenting stress. In addition, Sarant and Garrard (2014) found a significantly higher level of stress in parents of children with CIs and noted a negative correlation between parenting stress via the PSI-3 and DHH children’s language abilities. A study employing a context-specific parenting stress measure for families of children with hearing loss (e.g., Family Stress Scale) also found a significant negative association between parenting stress and spoken language outcomes in children three years following cochlear implantation by way of parental self-efficacy (Cejas et al., 2021). These studies highlight that parenting stress may serve as a significant risk factor for DHH children’s language outcomes, even at moderate levels.

Although parenting stress is known to affect interactions between children and their caregivers (Coplan et al., 2003), it has not been examined in relation to child temperament in DHH children. Examining how parenting stress can interact with child regulatory abilities and emotional reactivity highlights the reciprocal relationship between caregiver characteristics and child factors that jointly shape language development. Indeed, higher levels of parenting stress are associated with poorer social–emotional and language outcomes in young DHH children (mean age = 2;3; Dirks et al., 2016), highlighting the dynamic interplay among caregiver attributes, child attributes, and child language. Similar to interactions between parenting stress and child emotional skills, limited quality interactions between caregivers and children due to greater parenting stress may make it difficult for DHH children with less developed regulatory skills (i.e., effortful control) to achieve better language outcomes.

### 1.2. Rationale and research questions

The first purpose of the current study is to examine how caregiver language can affect the relation between effortful control and expressive language in school-aged DHH children. Previous work has shown associations between effortful control, caregiver language, and receptive language (Bowdrie et al., 2023). Expanding this work to investigate how caregiver–child interactions can affect expressive language might provide evidence that these interactions carry significance across many different areas of spoken language development beyond just receptive language. The second purpose of this study is to investigate how characteristics that strain caregiver–child interactions, specifically parenting stress, interacts with child effortful control to influence children’s expressive language. We predict that higher levels of effortful control in DHH children will support opportunities for language learning, and thus be positively associated with expressive language. Increased caregiver linguistic input may help to further scaffold expressive language in DHH children whereas increased parenting stress may constrain the quality of these caregiver–child interactions, impacting child expressive language outcomes.

In this study, we focused on expressive communicative language – the use of expressive communication skills in the context of social communication – in contrast to expressive language ability – the individual’s capacity of expressive language knowledge or discourse skills. Expressive communicative language is measured in the context of naturalistic social language interactions, whereas expressive language ability is measured with normed lab-based ability tests such as expressive vocabulary tests. Expressive communicative language more closely approximates the quality of language use in real-world environments and interactions and is therefore a critical functional outcome and goal for DHH children.

## 2. Method

### 2.1. Participants

DHH children and a primary caregiver were recruited from a larger, longitudinal study examining developmental outcomes in DHH children with HAs and CIs who were between the ages of 3;0–8;11 at the beginning of the study. The current sample included a subset of 59 families with DHH children aged 3;0 to 7;11 at the start of the study. This age range was selected because our measure of child effortful control – the Child Behavior Questionnaire – Short Form (Putnam & Rothbart, 2006) – was normed on children within this age range. Families were primarily recruited from Ohio and Indiana. All families identified spoken language as a goal for their child and English as the primary language spoken in the home. Children passed a nonverbal cognitive screener (score greater than 2 SD below the mean), the Picture Similarities subtest of the Differential Ability Scales-II (Elliot, 2007), and did not have a diagnosis of auditory neuropathy or developmental disorders (other than those associated with their hearing loss). Primary caregivers had no reported hearing difficulties. Demographics for children and their caregivers are shown in Table 1.

**Table 1.** Child and caregiver demographics for DHH group (*n* = 59) and hearing aid (*n* = 25) and cochlear implant (*n* = 34) subgroups

DHH child characteristics		HA subgroup	CI subgroup
Age, years; months	*M* = 5;9 *SD* = 1;4	*M* = 5;6 *SD* = 1;3	*M* = 5;11 *SD* = 1;4
Gender^a^, *n* female/male/nonbinary	28/31/0	13/12/0	15/19/0
Race, *n*			
Asian	3	1	2
Bi- or multiracial	4	2	2
Black or African American	6	4	2
White	46	18	28
Ethnicity, *n*			
Hispanic or Latino/a	2	1	1
Not Hispanic or Latino/a	57	24	33
Length of device use^b^, years;months	*M* = 4;5 *SD* = 1;7	*M* = 4;7 *SD* = 1;9	*M* = 4;3 *SD* = 1;6
Aided Better-Ear Pure-Tone Average^c^, dB HL	*M* = 25.01 *SD* = 7.68	*M* = 22.97 *SD* = 15.48	*M* = 25.60 *SD* = 3.56
Unaided Better-Ear Pure-Tone Average^d^, dB HL	*M* = 53.16 *SD* = 18.99	*M* = 47.00* *SD* = 14.17	*M* = 80.25* *SD* = 12.73
*Caregiver characteristics*			
Highest level of education^e^	Partial 4-year college	Partial 4-year college	Partial 4-year college
Annual household income^f^	$65,000–$79,999	$65,000–$79,999	$65,000–$79,999
Caregiver relationship			
Mother	54	24	30
Father	3	1	2
Grandmother	2	0	2

*Note:* CI, cochlear implant; DHH, deaf or hard of hearing; HA, hearing aid.

a “Nonbinary” category not included in chi-squared analysis due to having a value of “0”.

b Calculated from when child was first fit with HA or CI.

c Subset of sample from which data was available (DHH Group = 36; HA Subgroup = 8, CI Subgroup = 28).

d Subset of sample from which data was available (DHH Group = 27; HA Subgroup = 22, CI Subgroup = 5).

e Scored on a 10-point interval.

f Scored on a 10-point ordinal scale.

* *p* < .001.

The sample included 25 children with bilateral HAs who were diagnosed with hearing loss and fit with HAs by 3;0 (92% by 2;0) and 34 children with CIs (32 with bilateral CIs, 2 with bimodal CI/HA) who were diagnosed and received their CI by 3;6 (88% by 2;0 years of age). The HA and CI subgroups did not differ in age, *t*(57) = −1.432, *p* = .158; gender, χ^2^ (1) = 0.359, *p* = .549; length of device use, *t*(57) = .852, *p* = .398; better-ear aided pure-tone average (PTA), *t*(34) = −.850, *p* = .401; highest level of caregiver education, *t*(57) = .211, *p* = .834; or average household income *t*(57) = .841, *p* = .404. The only demographic factor that the subgroups differed in was unaided better-ear PTA, *t*(25) = −4.81, *p* < .001, as expected. Therefore, children with HAs and CIs were collapsed into one group to increase power for analyses. It should be noted that audiologic data were collected for a subset of DHH children (see Table 1), following several attempts to obtain these data.

### 2.2. Materials

#### 2.2.1. Effortful control

The Child Behavior Questionnaire–Short Form (CBQ-SF; Putnam & Rothbart, 2006) is a 94-item parent-report questionnaire that assesses temperament in children between the ages of 3 and 7 years across 15 subscales that form three factors: effortful control, negative affectivity, and surgency-extraversion. Caregivers rate each item on a Likert scale on how likely it is for their child to respond a certain way in different situations, ranging from 1 (extremely untrue) to 7 (extremely true). We assessed children’s inherent regulatory skills using the effortful control construct, which is defined as the child’s ability to leverage attentional resources and wilfully regulate emotions and behaviours (Rothbart et al., 2003). Effortful control is made up of the attentional focusing, inhibitory control, low-intensity pleasure, and perceptual sensitivity subscales of the CBQ-SF, all of which show fairly good internal consistency (between 0.69–0.75).

#### 2.2.2. Parenting stress

The Parenting Stress Index–Fourth Edition Short Form (PSI-4-SF; Abidin, 2012) is a widely used 36-item measure that assesses the level of stress stemming from parenting roles in families with children from birth to 12 years old. Caregivers indicate the extent to which they agree with items related to the caregiver–child system on a scale of 1 (strongly disagree) to 5 (strongly agree). Each item loads onto one of three domains – parental distress, parent–child dysfunctional interaction, and difficult child – that together make up a total parenting stress score. All three domains and the total stress score of the PSI-4-SF show good internal consistency (between 0.88–0.95; Abidin, 2012). This total score (i.e., the sum raw score of all items ranging from 36 to 180) was used for analyses. Higher scores on the PSI-SF-4 are indicative of greater levels of parenting stress.

#### 2.2.3. Caregiver language

Primary caregivers’ language was quantified using the number of different word (NDW) roots and mean length of utterance (MLU) in morphemes used by the caregivers during a dyadic play interaction with their DHH child. The Systematic Analysis of Language Transcripts–Version 2 (SALT-2; Miller & Iglesias, 2012) software was used to transcribe language samples derived from the play interaction. NDW, which captures the richness of vocabulary used by caregivers during the interaction, and MLU, which reflects the variety of grammatical forms, were obtained from analyses performed in SALT-2. Each caregiver’s NDW and MLU were standardised through Z transformation and averaged together to create a composite score of “Caregiver Language,” which was used for analyses.

#### 2.2.4. Child expressive language

DHH children’s language was also obtained from the dyadic play interaction, and transcribed and analysed using SALT-2 software. To remain consistent with caregiver language variables – and to build on findings from Bowdrie et al. (2023), which incorporated receptive language outcomes using standardised assessments in a similar sample of DHH children – we focused on NDW and MLU as measures of expressive language in DHH children. A composite of child NDW and MLU was formed through averaging Z scores of each child’s NDW and MLU used during the interaction. This composite score of “Child Expressive Language” was used for analyses.

### 2.3. Procedures

The Ohio State University Institutional Review Board approved all research performed with DHH children and their caregivers. Data were collected as part of the larger, longitudinal study during 1.5- to 2-hour home visits. Prior to each home visit, caregivers were provided with regulatory forms (i.e., consent, parental permission for child) and questionnaires, including the CBQ-SF and the PSI-4-SF, by mail. At the time of the home visit, two trained examiners were present: one worked with the child to complete a battery of language and neurocognitive assessments, while the other examiner worked with the primary caregiver to review previously mailed materials and to complete additional questionnaires and testing with the caregiver. Note that measures discussed in this paper reflect only a subset of data collected as part of the larger study.

Towards the end of the home visit, DHH children and their caregiver engaged in a video recorded, semi-structured play interaction. Examiners provided each dyad a set of age-appropriate toys and recorded the interaction using a GoPro Hero4 video camera that was connected to omnidirectional microphones attached to both the child and their caregiver. The session included 15 minutes of the child and caregiver engaging in play (families were instructed to play together as normal) and concluded with an additional 5-minute clean-up session in which the child was required to put away the toys. Caregivers were not allowed to physically help with cleaning up the toys, but they could provide verbal assistance to their child. Language data (i.e., MLU and NDW) were obtained for each DHH child and caregiver from transcripts using a standard measure report from SALT-2 and were based solely on the 15-minute play interaction (the 5-minute clean-up session was not included). Two trained research assistants transcribed and coded the language of caregivers and children from the videos of play interactions in SALT-2. A senior research assistant, who was also a certified speech-language pathologist, checked for discrepancies between coders and made the final determination based on video content.

## 3. Results

### 3.1. Descriptive statistics of child and caregiver variables

Table 2 displays descriptive data for DHH children’s effortful control and expressive language, and caregivers’ parenting stress and language use. Caregivers’ ratings of DHH children’s effortful control were similar to previous studies that have examined the relation between temperament and spoken language outcomes in this clinical population (Bowdrie, et al. 2022, 2023), which is not surprising as some of the participants in this study also were in those studies. There were not significant differences in effortful control ratings between the boys and girls, *t*(57) = .721, *p* = .474, which differs from results on typically hearing children, which tend to reveal sex differences (Else-Quest et al., 2006). The measurements comprising the expressive language composite for DHH children (i.e., MLU and NDW) showed wide variability, consistent with literature examining expressive language outcomes in this population (Niparko et al., 2010; Nittrouer et al., 2020). On average, DHH children in our sample had an MLU of 3.16 morphemes, which is below what would be expected for typically developing children (i.e., 4.96) within the same average age of our sample (5;6–5;11 years; Rice et al., 2010). It is a bit more difficult to compare NDW to normative data, as this measure can vary based on the length of the language sample, but research tends to support that this measurement is associated with MLU (Dethorne et al., 2005; Hewitt et al., 2005). Caregivers’ average level of MLU (i.e., 4.39) and NDW (i.e., 208.46) used during the play interaction was higher than what was observed in DHH children, as expected. Additionally, caregivers’ MLU and NDW ranges were wide, which may be due in part to various mechanisms that have been examined in the literature, such as responsivity or caregivers adapting to their child’s age and/or language abilities (Ambrose et al., 2015, Curtin et al., 2021) during the play interaction. Indeed, children’s age varied across a 5-year age range, which likely partially contributed to the variability in caregivers’ MLU and NDW.

**Table 2.** Descriptive data for child and caregiver measures, including mean, standard deviation, and range

	M *(SD)*	Range
CBQ-SF: Effortful control	5.13 *(0.65)*	3.69–6.29
Child expressive language composite score (Z-score)	0.00 *(0.93)*	−1.84–1.95
Child expressive language: MLU	3.16 *(0.92)*	1.45–5.06
Child expressive language: NDW	129.12 *(44.37)*	42.00–241.00
PSI-SF–4: Parenting stress	68.95 *(21.90)*	38.00–129.00
Caregiver language composite score (Z-score)	0.00 *(0.89)*	−1.53–2.44
Caregiver language: MLU	4.39 *(0.72)*	3.01–6.44
Caregiver language: NDW	208.46 *(44.57)*	105.00–332.00

*Note:* MLU, mean length of utterance (morphemes); NDW, number of different words (unique word roots, excluding affixes).

Considerable variability was also observed in reported parenting stress. Recall that the possible range of scores for the PSI-SF-4 is 36–180. The normal range of parenting stress on the PSI-SF-4 span the 16^th^ to 84^th^ percentiles (raw score of 54 to 109). The average PSI-SF-4 score in our sample was 68.95, which corresponds to the 42^nd^ percentile (i.e., within the normal range). About 27% of caregivers reported parenting stress below the 16^th^ percentile (i.e., a total score of 54 or less), while 5% of caregivers reported parenting stress exceeding the 84^th^ percentile (i.e., a total score of 109 or more).

### 3.2. Relations between child effortful control, child expressive language, caregiver language, and parenting stress

Partial Pearson correlations were performed in SPSS-version 29.0 (IBM Corp, 2023) to examine the relations between child effortful control, child expressive language, caregiver language, and parenting stress while controlling for caregiver education level, which is known to contribute to DHH children’s language development (Tomblin et al., 2015). The results are presented in Table 3. Child effortful control was negatively related to parenting stress, *r*(56) = −.470, *p* < .001. In addition, child and caregiver language production were positively correlated with each other, *r*(56) = .495, *p* < .001. Of note, child expressive language was significantly and positively correlated with child age, *r*(56) = .408, *p* = .001, as expected, whereas caregiver language was not significantly associated with child age, *r*(56) = .022, *p* = .869. While not significant, there was a trend for greater parenting stress to be associated with lower caregiver language, *r*(56) = −.220, *p* = .097. All other correlations were not statistically significant.

**Table 3.** Partial Pearson correlations between child and caregiver variables (controlling for caregiver education)

Measure	1	2	3	4
1. CBQ-SF: Child effortful control	-			
2. Child expressive language composite score (Z-score)	.145	-		
3. PSI-SF-4: Parenting stress	−.470**	.053	-	
4. Caregiver language composite score (Z-score)	.214	.495**	−.220	-

*Note:* degrees of freedom = 56

** *p* < .001.

### 3.3. Moderating effects of parenting stress on child effortful control and child expressive language

**Figure 1.**
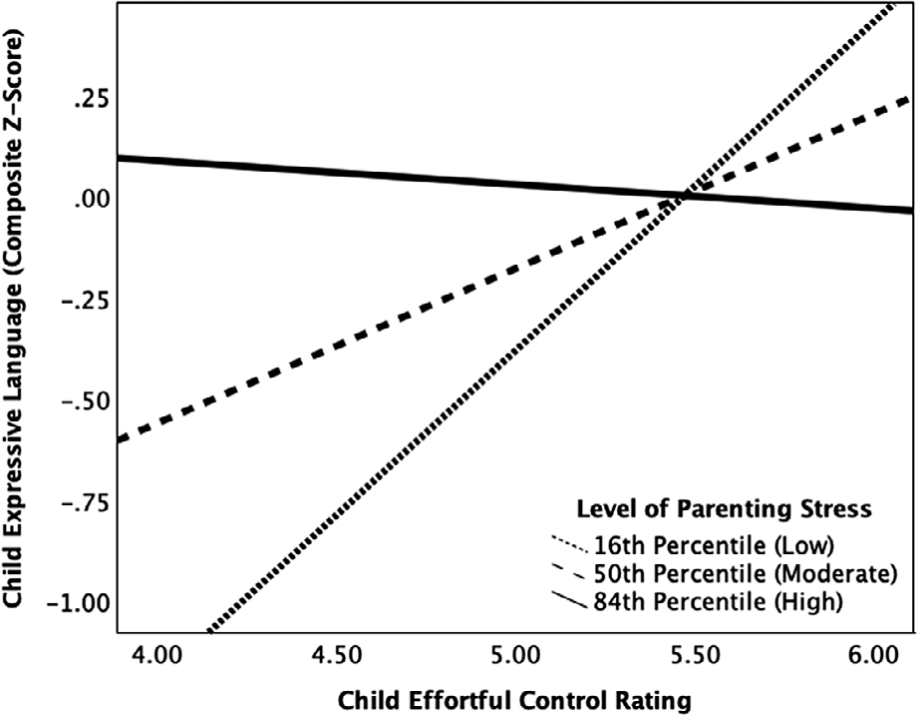
The relation between child effortful control and expressive language at the 16^th^ (low), 50^th^ (moderate), and 84^th^ (high) percentiles of parenting stress within the sample.

Moderation analyses were performed using the PROCESS SPSS macroinstruction developed by Hayes (2022). First, we ran a regression model predicting child expressive language from child effortful control, parenting stress, the interaction between child effortful control and parenting stress (which is the product of these two variables), and caregiver education as a covariate, as shown in Table 4. Although child effortful control did not significantly correlate with child expressive language, moderation analyses can still be performed to unveil the conditional effects between these two variables based on a third possible variable (e.g., parenting stress), especially when moderation is theoretically indicated (Hayes, 2022). While the regression model was marginally significant, *R^2^* = 0.39, *F*(4, 54) = 2.43, *p* = .059, child effortful control and parenting stress were shown to be significant predictors of child expressive language. Additionally, the interaction term (i.e., child effortful control x parenting stress) within this model was significant, *F*(1, 54) = 5.17, *p* = .027), Δ*R^2^* = .08, indicating that the relation between child effortful control and child expressive language was moderated by parenting stress. Despite the overall model being only marginally significant, PROCESS probes a significant interaction term to reveal potentially meaningful interaction effects that is otherwise lost in multiple linear regression models (Preacher & Hayes, 2004). To further probe the interaction between these variables, we used PROCESS to identify the 16^th^ (low), 50^th^ (moderate), and 84^th^ (high) percentiles of parenting stress based on our sample (not the percentiles established by the measure itself). At the 16^th^ percentile of our sample’s reported parenting stress, child effortful control positively related to child expressive language (*b* = .81; *p* = .009). However, at the 50^th^ (*b* = .38; *p* = .099) and 84^th^ percentiles (*b* = −.06; *p* = .822) of parenting stress within our sample, child effortful control no longer was related to child expressive language. The conditional relation between child effortful and child expressive language at these different levels of parenting stress are displayed in Figure 1. Further probing of this significant interaction using the Johnson-Neyman approach in PROCESS revealed significant moderation between effortful control and expressive language at levels of parenting stress at or below scores of 65.30 *(b* = .43, *p* = .045), which includes 47% (*n* = 28) of families within our sample.

**Table 4.** Results of regression model predicting child expressive language from child effortful control, parenting stress, their interaction, and caregiver education (as a covariate)

	*β*	*SE*	*t*	*p*
Child effortful control	1.61	.61	2.64	.010*
Parenting stress	.10	.04	2.40	.019*
Child effortful control x parenting stress	−.02	.01	−2.27	.026*
Caregiver education (covariate)	.09	.08	1.13	.263
*Model Summary: R^2^* = 0.39, *F*(4, 54) = 2.43				.059

* *p <* .05.

### 3.4. Moderating effects of caregiver language on child effortful control and child expressive language

**Figure 2.**
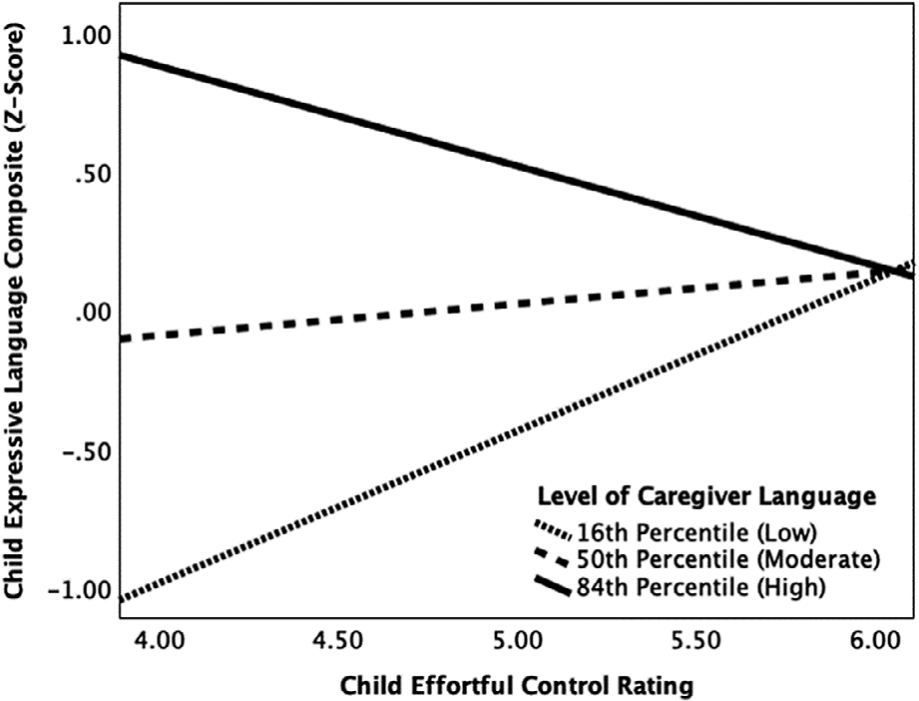
The relation between child effortful control and expressive language at the 16^th^ (low), 50^th^ (moderate), and 84^th^ (high) percentiles of caregiver language within the sample.

A second moderation analyses was performed to examine the effect of caregiver language on the relation between child effortful control and expressive language. The regression model (shown in Table 5) with child effortful control, caregiver language, their interaction (i.e., child effortful control x caregiver language) and caregiver education as a covariate predicting child expressive language was significant, *R^2^* = .33, *F*(4, 54) = 6.75, *p* < .001. Caregiver language and the product of child effortful control and caregiver language, *F*(1, 54) = 5.05, *p* = .029, Δ*R^2^* = .06, contributed significantly to this model. At the 16^th^ percentile, or when caregivers’ language level was low, child effortful control was positively related to child expressive language (*b* = .55; *p* = .049). When caregivers’ language level was moderate (50^th^ percentile; *b* = .11, *p* = .495) or high (84^th^ percentile; *b* = −.36, *p* = .151), child effortful control was not related to child expressive language. Figure 2 displays the moderating effects of caregiver language at these different percentiles. Further, a Johnson-Neyman analysis revealed that significant moderating effects of caregiver language on the relation between child effortful control and expressive language occurred at or below composite caregiver language z-scores of −1.13 (*b* = .63, *p* = .041), which included about 15% of the sample (*n* = 9). Moderating effects of caregiver language also occurred at or above caregiver language z-scores of 2.24 (*b* = −.99, *p* = .049), reflecting only about 2% of our sample (*n* = 1).

**Table 5.** Results of regression model predicting child expressive language from child effortful control, caregiver language, their interaction, and caregiver education (as a covariate)

	*β*	*SE*	*t*	*p*
Child effortful control	.08	.16	.51	.611
Caregiver language	2.92	1.08	2.71	.009*
Child effortful control x caregiver language	−.48	.21	−2.24	.029*
Caregiver education (Covariate)	.07	.07	.97	.337
*Model summary: R^2^* = .33, *F*(4, 54) = 6.75				<.001**

* *p < .*05,

** *p < .*001.

## 4. Discussion

Child–caregiver interactions reflect a foundational dynamic for children’s language, psychosocial, and behavioural development (Christakis et al., 2019; Gadaire et al., 2017; Hollenstein et al., 2004), and are especially important for DHH children who are at risk for developmental delays (Curtin et al., 2021; Nittrouer et al., 2020). Previous research in a similar sample of DHH children revealed that greater levels of lexical diversity from caregivers allowed all DHH children–regardless of their level of effortful control – to achieve optimal receptive language outcomes (Bowdrie et al., 2023). The current study sought to examine the interactive effects on another dimension of child language development – expressive language in naturalistic communication – in a similar sample of DHH children and caregivers. Replicating and extending these results to another domain of language development helps validate and generalise the earlier findings. In addition, the current study examined how an additional parental characteristic that can affect child–caregiver interactions – parenting stress – might interact with effortful control to affect child expressive language.

Prior to moderation analyses, we examined the associations between child effortful control, caregiver language, parenting stress, and child language. We found a positive association between the composite measures of child expressive language and caregiver language to their child, which is consistent with the literature on DHH children (DesJardin & Eisenberg, 2007; Yoshinaga-Itano et al., 2020). We also found that caregivers who rated their children higher in effortful control on the CBQ-SF reported lower levels of parenting stress on the PSI-4-SF. Recall that effortful control reflects the capacity to wilfully adapt attention, behaviour, and emotions (Eisenberg et al., 2004; Rothbart et al., 2003). One possible reason for this negative association may be that children who exhibit greater abilities for regulating emotions and behaviours contribute to more positive caregiver-child interactions, and thus less stress related to parenting. On the other hand, children with less regulatory abilities may be perceived as inattentive and highly reactive, which can increase parenting demands and lead to greater parenting stress. The relation between child effortful control and parenting stress likely is bidirectional in that greater parenting stress can impact a child’s ability to leverage effortful control skills (Yıldız & Uzundağ, 2024). Indeed, studies have shown that decreased responsivity and other disengaged parenting practices that are associated with parenting stress do not provide a constructive model for children to develop effective self-regulatory skills (Deater-Deckard, 2008; Neece et al., 2012).

We were surprised by the lack of direct associations between parenting stress and both caregiver and child language. Higher levels of parenting stress impose challenges within the caregiver–child dyad and have shown negative associations with child emotional and behavioural outcomes (Barroso et al., 2018; Deater-Deckard, 2008). However, less attention has been given to how parenting stress can affect caregiver language input to children, especially DHH children. Parenting stress is associated with more authoritative parenting styles, less caregiver involvement, greater degrees of depression and anxiety in caregivers, and more (Fang et al., 2021; Khalsa et al., 2022). These characteristics can affect caregivers’ quality and quantity of linguistic input to DHH children. For example, authoritarian parenting styles may prompt greater use of directives and other forms of communication that are less facilitative in nature. In addition, less involved caregivers likely provide fewer opportunities for language learning for children. As a result, we expected parenting stress to be negatively associated with both the caregiver and child language measures. Although there was a trend for greater parenting stress to be associated with less lexical diversity and syntactic complexity in caregiver language in this study, this relationship was not significant. The absence of these associations may be due to the context through which our caregiver and child language measures were derived. That is, although we instructed caregiver–child dyads to engage in a play interaction as they normally would, this context may have not been sensitive enough to capture the effects parenting stress has on caregiver language use with children. In other words, perhaps other styles of interaction outside of the play realm might reveal the effects of parenting stress of caregiver language use with children, such as language use during a goal-oriented task such as getting ready for school. In addition, the average level of parenting stress reported by caregivers within our sample was within the typical range, which could account for the lack of association between caregiver language and parenting stress. The effects of parenting stress on caregivers’ language may become more apparent in families of DHH children who report greater (i.e., clinically significant) levels of parenting stress.

### 4.1. Moderating influence of caregiver language on the relation between effortful control and expressive language

The first purpose of the study was to examine the interactive effect of child effortful control and caregiver language input on expressive language in DHH children. Although there was a lack of a direct association between child effortful control and child expressive language, our moderation analysis revealed that the significance of this relationship depended on the level of caregiver language input. When caregivers provided less language input to DHH children during the play interaction, there was a positive relationship between child effortful control and child expressive language. That is, in language environments with limited linguistic input from caregivers, only DHH children with greater regulatory skills showed better expressive language. However, when caregivers had moderate to high levels of input to DHH children during the play interaction, child effortful control no longer was related to child expressive language. These findings highlight that DHH children with lower levels of effortful control may show some resiliency in terms of their expressive language development but only when caregivers are providing at least moderate levels of linguistic input. Although greater effortful control is thought to facilitate child language development through regulatory abilities that support the processing of linguistic input (Bloom, 1993; Davison et al., 2019; Dixon & Smith, 2000), increased levels of caregiver input appear to be a way for DHH children with poorer effortful control to compensate for suboptimal regulatory skills. This aligns with previous research that revealed more complex regulatory skills (i.e., inhibitory control) in DHH children are associated with caregivers’ use of higher-level linguistic input such as mental state language (Lind-Combs & Holt, 2022), which may further underscore how caregivers can scaffold developmental outcomes in DHH children through their linguistic input.

### 4.2. Moderating influence of parenting stress on the relation between effortful control and expressive language

The second purpose of the study was to examine the interactive effect of child effortful control and parenting stress on expressive language in DHH children. Although the moderation model predicting the interactive effect of effortful control and parenting stress on child expressive language was only marginally significant, there is evidence that greater levels of parenting stress were negatively associated with expressive language in DHH children, despite their regulatory skills. In this model, when caregivers reported lower parenting stress, greater effortful control was related to greater expressive language. This relationship was not observed in families who reported moderate to high levels of parenting stress within the sample. These trends seem to provide evidence that greater parenting stress can pose a risk for DHH children in terms of their expressive language development. While the mechanisms through which parenting stress affects language outcomes in DHH children have not been thoroughly explored, it is clear that parenting stress affects caregiver-child interactions. As discussed above where we observed a trend between parenting stress and caregiver language, a likely mechanism may actually be that increased parenting stress negatively affects language learning opportunities by affecting the quality and quantity of caregivers’ language. Though more work is needed to explore these relations between child effortful control, parenting stress, caregiver language, and child language, a recent study revealed that family environments with high levels of chaos within the physical home environment appear to interfere with the ability for school-age DHH children to achieve optimal language comprehension skills (Bowdrie et al., 2022). Given the known association between household chaos, caregiver stress, and caregiver language (Coldwell et al., 2006; Evans & Wachs, 2010), this recent study may highlight an important contextual pathway through which caregiver stress and environmental factors together can affect opportunities for language learning in DHH children. There is a need for future research to unpack how individual child characteristics (e.g., effortful control), caregiver factors (e.g., domain-specific stress, linguistic input), and broader environmental influences interact to shape language development.

### 4.3. Clinical implications

Interventions for supporting families with DHH children who have goals of spoken language development for their children have recognised the importance of the family environment to developmental outcomes. Indeed, including families and providing social supports have been implemented in interventions for years. The current study provides further support for the inclusion of families into interventions, with an emphasis on empowering caregivers to leverage their language input to support their DHH children. This appears particularly important for DHH children who present with inherent abilities that may seem to be barriers to language learning.

### 4.4. Limitations

When examining child–caregiver interactions, it is important to acknowledge the bidirectional influences that occur. The study is cross-sectional and thus did not explore longitudinal relationships between the effects of child effortful control and caregiver language on DHH children’s language to support the direction of these influences. However, we have employed theories of temperament to support child temperament as a predictor of language in this clinical population that is influenced by caregiver characteristics, given the biological nature of this child characteristic. However, we recognise that child temperament may serve as a moderating influence within certain transactional contexts. Lastly, we noted a significant positive association between child age and expressive language in DHH children. The current study did not account for age in subsequent statistical analyses as the authors did not identify an *a priori* reason to control for age. However, supplemental analyses not reported here revealed that including age as a covariate did not alter the pattern of results or interpretation of the moderation model that examined the effect of the interaction between child effortful control and caregiver language on child expressive language. In the model including the interaction of child effortful control and parenting stress, adding age as a covariate slightly affected the *p*-values, though these findings were difficult to interpret given the small effect sizes and reduction in statistical power. Future research with a larger sample should examine whether age significantly affects the interactive relationship between these child and caregiver characteristics.

## 5. Conclusion

The interactions that occur between children and their caregiver(s) are important dynamics to examine in relation to language development, especially in DHH children who can experience wide variability in spoken language development. The current study provides evidence that greater levels of caregiver linguistic input can help even DHH children who exhibit lower levels of effortful control to achieve optimal expressive language outcomes. Moreover, although only marginally significant, it appears that even moderate levels of parenting stress in caregivers of DHH children can affect the ability of even those with strong regulatory skills to achieve better expressive language. This work provides further evidence that caregiver language input to DHH children may serve as a mechanism that can enable this clinical population to leverage their inherent regulatory abilities to achieve better spoken language outcomes. Furthermore, there is evidence that parenting stress can strain the quality of caregiver–child interactions, which in turn can negatively impact the relation between child effortful control and expressive language.
